# Carrot Juice Fermentations as Man-Made Microbial Ecosystems Dominated by Lactic Acid Bacteria

**DOI:** 10.1128/AEM.00134-18

**Published:** 2018-05-31

**Authors:** Sander Wuyts, Wannes Van Beeck, Eline F. M. Oerlemans, Stijn Wittouck, Ingmar J. J. Claes, Ilke De Boeck, Stefan Weckx, Bart Lievens, Luc De Vuyst, Sarah Lebeer

**Affiliations:** a University of Antwerp, Research Group Environmental Ecology and Applied Microbiology (ENdEMIC), Department of Bioscience Engineering, Antwerp, Belgium; b Vrije Universiteit Brussel, Research Group of Industrial Microbiology and Food Biotechnology (IMDO), Faculty of Sciences and Bioengineering Sciences, Brussels, Belgium; c Laboratory for Process Microbial Ecology and Bioinspirational Management (PME&BIM), Department of Microbial and Molecular Systems (M2S), KU Leuven, Campus De Nayer, Sint-Katelijne-Waver, Belgium; University of Bayreuth

**Keywords:** 16S RNA sequencing, RNA-based 16S amplicon sequencing, amplicon sequence variants, biogenic amines, fermentation, lactic acid bacteria, phylogenetic placement

## Abstract

Spontaneous vegetable fermentations, with their rich flavors and postulated health benefits, are regaining popularity. However, their microbiology is still poorly understood, therefore raising concerns about food safety. In addition, such spontaneous fermentations form interesting cases of man-made microbial ecosystems. Here, samples from 38 carrot juice fermentations were collected through a citizen science initiative, in addition to three laboratory fermentations. Culturing showed that Enterobacteriaceae were outcompeted by lactic acid bacteria (LAB) between 3 and 13 days of fermentation. Metabolite-target analysis showed that lactic acid and mannitol were highly produced, as well as the biogenic amine cadaverine. High-throughput 16S rRNA gene sequencing revealed that mainly species of Leuconostoc and Lactobacillus (as identified by 8 and 20 amplicon sequence variants [ASVs], respectively) mediated the fermentations in subsequent order. The analyses at the DNA level still detected a high number of Enterobacteriaceae, but their relative abundance was low when RNA-based sequencing was performed to detect presumptive metabolically active bacterial cells. In addition, this method greatly reduced host read contamination. Phylogenetic placement indicated a high LAB diversity, with ASVs from nine different phylogenetic groups of the Lactobacillus genus complex. However, fermentation experiments with isolates showed that only strains belonging to the most prevalent phylogenetic groups preserved the fermentation dynamics. The carrot juice fermentation thus forms a robust man-made microbial ecosystem suitable for studies on LAB diversity and niche specificity.

**IMPORTANCE** The usage of fermented food products by professional chefs is steadily growing worldwide. Meanwhile, this interest has also increased at the household level. However, many of these artisanal food products remain understudied. Here, an extensive microbial analysis was performed of spontaneous fermented carrot juices which are used as nonalcoholic alternatives for wine in a Belgian Michelin star restaurant. Samples were collected through an active citizen science approach with 38 participants, in addition to three laboratory fermentations. Identification of the main microbial players revealed that mainly species of Leuconostoc and Lactobacillus mediated the fermentations in subsequent order. In addition, a high diversity of lactic acid bacteria was found; however, fermentation experiments with isolates showed that only strains belonging to the most prevalent lactic acid bacteria preserved the fermentation dynamics. Finally, this study showed that the usage of RNA-based 16S rRNA amplicon sequencing greatly reduces host read contamination.

## INTRODUCTION

Food fermentations represent humankind's first venture in biotechnology, applied primarily for preservation purposes and the generation of bioactive metabolites (1, 2). Recently, spontaneous household food fermentations have regained popularity among chefs and nonprofessional home fermentation enthusiasts, not only for the postulated digestive and other health benefits of fermented foods, but also for their rich and unique flavors (3–5). Fermentation of vegetables is especially appealing, since vegetables are a source of essential nutrients, vitamins, minerals, antioxidants, and fibers and have a low sugar content (6, 7). In addition, certain strains of fermentative lactic acid bacteria (LAB), which often belong to species of the genus Lactobacillus, could have health-promoting effects, for instance, through immunomodulation and pathogen inhibition (4, 8–10).

Despite the fact that an increased intake of fermented foods clearly has potential benefits, some concerns have been raised regarding the food safety of artisan fermented foods, since spontaneous fermentations first require in-depth knowledge of the fermentation process and how to control it to avoid food poisoning (11). Compared to industrial dairy, meat, and alcoholic fermentations, little is known about the microbial characteristics and community dynamics of spontaneous fermentation processes in general and spontaneous vegetable fermentations in particular (3, 12, 13). Except for the well-known sauerkraut, kimchi, and cucumber fermentations, spontaneous vegetable fermentation processes encompass carrots, cauliflower, leek, olives, tomatoes, and a mixture of those, which are served as both main and side dishes (14–17). In Belgium, fermented carrot juice is offered as an alternative drink to wine at some Michelin star restaurants, and this was the inspiration for the current study.

Although previous studies have already described the use of starter-culture strains in carrot juice fermentations, none of them described the microbial community dynamics of the spontaneous fermentation process (6, 18–21). The aim of the present study was to obtain detailed insights into the microbial community dynamics, substrate consumption, and metabolite production kinetics of this man-made microbial ecosystem and evaluate its usage as a model system for studying LAB diversity. Therefore, the robustness and microbial composition of 2 laboratory- and 38 home-fermented carrot juices, collected through a citizen science project, were evaluated with culture-dependent plating and high-throughput amplicon sequencing. As a discrepancy between these data was found, a third laboratory fermentation was set up to identify the metabolically active drivers. Furthermore, to obtain more insights into the diversity of the key players in this ecosystem, their phylogenetic distribution was assessed across the Lactobacillus genus complex. Finally, as these key players belonged to different phylogenetic groups, their impact on the microbial dynamics of the fermentation process was evaluated and compared with two allochthonous Lactobacillus strains originally isolated from two human niches.

## RESULTS

### Carrot juice fermentations represent a robust process dominated by lactic acid bacteria.

**FIG 1:**
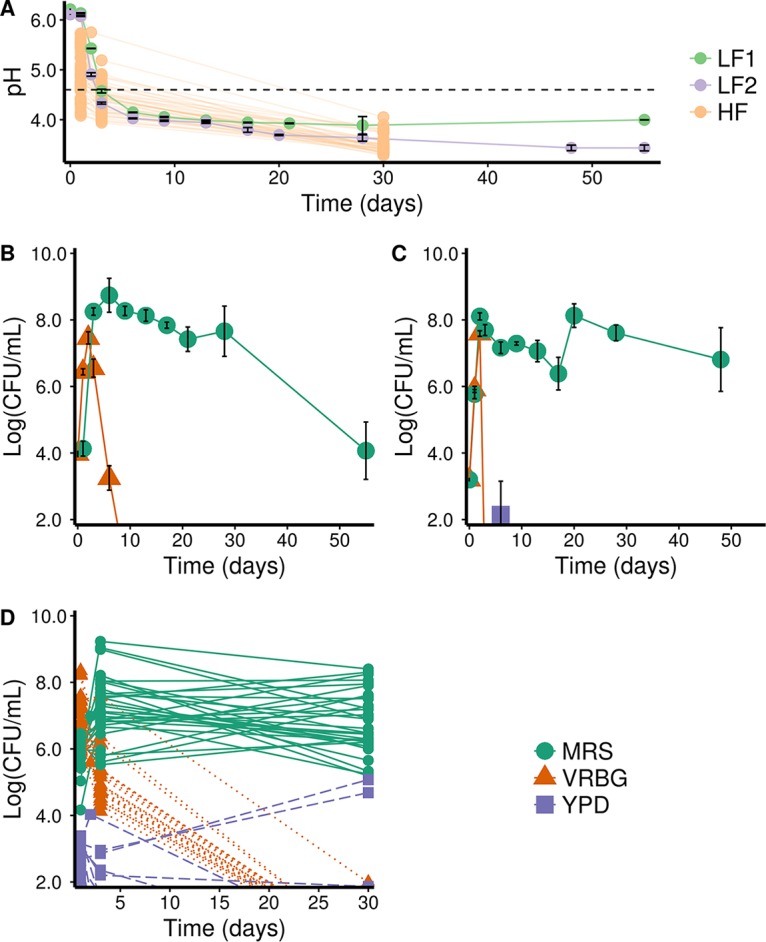
pH and microbial community dynamics of spontaneous carrot juice fermentations. (A) Evolution of the mean pH of biological repeats and standard errors of means of two laboratory fermentations of carrot juice (LF1 in light green and LF2 in purple) and of the pH of all household fermentations (HF; orange). The dashed horizontal line indicates the widely accepted food fermentation safety threshold of pH 4.6 (22). Data points of the same fermentation were connected for clarity. (B to D) Evolution of the mean microbial counts of biological repeats and standard errors of means on MRS agar for presumptive LAB, VRBG agar for presumptive Enterobacteriaceae, and YPD agar for presumptive yeasts, of LF1 (B), LF2 (C), and of the microbial counts of all household fermentations HF (*n* = 38) (D). Data points of the same fermentation were connected for clarity.

**FIG 2:**
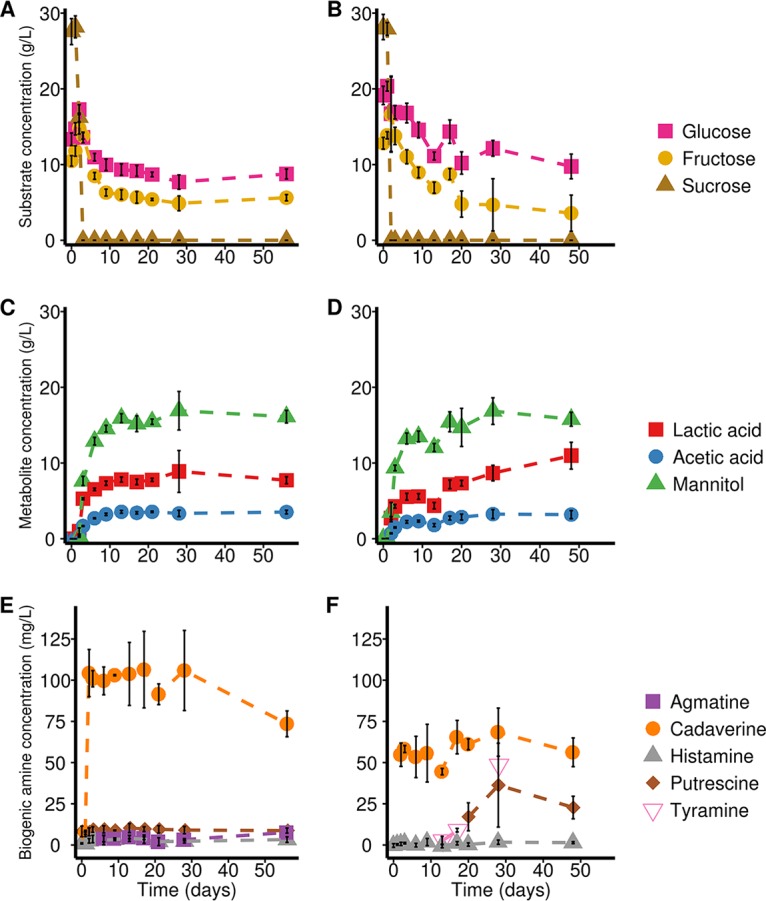
Substrate consumption and metabolite production profiles of the two laboratory carrot juice fermentations. (A and B) Residual substrate concentrations of LF1 (A) and LF2 (B). (C and D) Metabolite concentrations of LF1 (C) and LF2 (D). (E and F) Biogenic amine concentrations of LF1 (E) and LF2 (F). All data points represent the means of three biological repeats, and error bars represent standard deviations.

A citizen science carrot juice fermentation project allowed us to explore the robustness of 38 household fermentations (HF; three samples were not received), in particular regarding pH evolution (Fig. 1A) and microbial community dynamics (Fig. 1D), as well as microbial species diversity (see below). These household fermentations were used to account for various carrot sources (e.g., organic origin, supermarket, and own garden), kitchens, operators, and other environmental factors. In addition, two laboratory fermentations of carrot juice (LF1 and LF2) allowed a more in-depth time-series analysis of the pH evolution and microbiology (Fig. 1A to C), as well as substrate consumption and metabolite production (Fig. 2) of the fermented carrot juice microbiome, spanning a 2-month period.

After 2 months of fermentation, the average pH dropped from 6.2 to 4.0 for LF1 and from 6.1 to 3.4 for LF2. Similar trends were found for the household fermentations, for which the pH values ranged from 3.4 to 4.1 after 30 days of fermentation. The majority (72%) of the fermentations reached pH values lower than 4.6 at day 3, which is generally considered an important threshold from a food safety perspective (22). All fermentations reached a pH value below this threshold value at day 30 (Fig. 1A).

Selective plating on de Man-Rogosa-Sharpe (MRS) agar medium showed that high numbers of presumptive LAB (up to 10^9^ CFU/ml) were obtained from day 3 for all fermentations. LAB counts of the household fermentations varied between 2.67 × 10^5^ CFU/ml and 3.37 × 10^9^ CFU/ml at day 3 and between 1.33 × 10^5^ CFU/ml and 2.72 × 10^8^ CFU/ml at day 30 (Fig. 1D). Similar levels were obtained for both laboratory carrot juice fermentations, but counts decreased faster over time for LF1, leading to lower LAB counts of 1.17 × 10^4^ CFU/ml at the end of the experiment (Fig. 1B and C). This faster decline in LF1 possibly indicates an effect of carrot source on the fermentation process, since storage carrots were used for LF1, in contrast to the freshly harvested carrots used for LF2. Presumptive Enterobacteriaceae harboring potential foodborne pathogens were detected at the start of all fermentations. Whereas their cell density increased to approximately 10^7^ CFU/ml at day 3 and day 2 for LF1 and LF2, respectively, plate counts dropped to zero after 3 to 13 days of fermentation. Similar trends were found for the household fermentations, with high presumptive Enterobacteriaceae counts at the beginning of the fermentation, which dropped to zero in all cases, except for one (HF32) at day 30 (Fig. 1D). For this exception, a bad smell and presumed failure of the fermentation were reported by the citizen science participant. Presumptive yeasts did not prevail during these carrot juice fermentations, as no colonies could grow on yeast extract-peptone-dextrose (YPD) agar medium for the laboratory fermentations, except for a single time point in LF2 (Fig. 1C). In the household fermentations, higher numbers of presumptive yeast counts were detected at the first two time points, but these prevailed only in three fermentations, including again the failed fermentation HF32.

### Metabolite target analysis revealed high lactic acid and mannitol production in agreement with a LAB-dominated carrot juice fermentation process.

In addition to the bacterial community analysis, a metabolite target analysis was performed to assess carbohydrate consumption (Fig. 2A and B) and metabolite production (Fig. 2C and D) during the laboratory carrot juice fermentations LF1 and LF2. Sucrose was completely metabolized in both fermentations after 2 to 3 days. In contrast, incomplete consumption of glucose and fructose was found. Lactic acid and mannitol were identified as the main metabolites that were produced, with average end concentrations of 8.89 g/liter (LF1) and 10.97 g/liter (LF2) for lactic acid and 16.91 g/liter (LF1) and 16.86 g/liter (LF2) for mannitol. In addition, acetic acid was produced, albeit at low concentrations (Fig. 2C and D). These analyses thus further substantiated that the spontaneously fermented carrot juices were dominated by LAB, based on the high production of lactic acid. Moreover, the high levels of mannitol suggested the prevalence of heterofermentative LAB species, such as species of Leuconostoc.

In both laboratory carrot juice fermentations, the production of cadaverine was the highest of all biogenic amines detected. An increase in the cadaverine concentration occurred at day 2 for both fermentations, with average concentrations reaching up to 106.43 mg/liter for LF1 and 68.47 mg/liter for LF2. Since both presumptive Enterobacteriaceae and LAB showed high counts for that day, it is unclear which microorganisms were responsible for these high concentrations. In addition, agmatine, putrescine, and histamine were found in LF1, albeit at very low concentrations, reaching maximum average concentrations of 7.59, 10.29, and 4.00 mg/liter, respectively. No tyramine was found in this fermentation. In contrast, LF2 showed tyramine production, reaching a concentration of up to 48.63 mg/liter in one of the three biological repeats. Finally, LF2 contained a higher concentration of putrescine than LF1, reaching a maximum average concentration of 36.32 mg/liter on day 28.

### High-throughput 16S rRNA gene sequencing revealed Leuconostoc spp. and Lactobacillus spp. as the main LAB species.

High-throughput 16S rRNA gene sequencing (V4 region; Illumina MiSeq) resulted in 8,913,261 quality-controlled reads after data processing, using the recently developed fine-scale-resolution DADA2 pipeline (23). Most samples showed an observed amplicon sequence variant (ASV) (24) number between 10 and 50, whereas their Shannon diversity index varied between 1.2 and 2.5 (see Fig. S1 in the supplemental material). For all fermentations examined, one would expect a decrease in alpha diversity as a function of time, as the diversity at day 0 (which reflects the natural carrot microbial community) was likely to be higher than the diversity under the selective human-imposed fermentation conditions. However, following the removal of large amounts of contaminating host chloroplast DNA, relative low numbers of bacterial reads were obtained at the first time points. As a result, fewer bacterial taxa were detected at these sampling time points, probably explaining the trend of increasing diversity at the first time points, as found in most of the fermentations, except for LF1 (Fig. S1).

**FIG 3:**
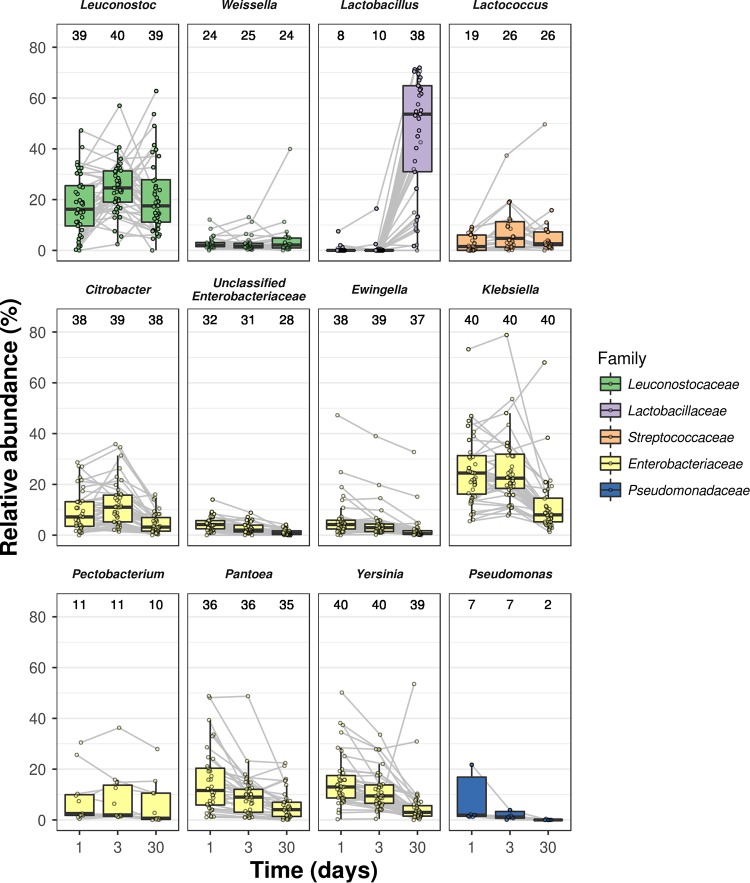
Taxonomic composition based on 16S rRNA gene sequencing (V4 region) of laboratory fermentations 1 (LF1) and 2 (LF2) and of the household fermentations (HF). The relative abundances of all amplicon sequence variants belonging to the same genus were summed, and only the 12 most abundant genera are shown. Relative abundances belonging to the same fermentation are connected with a gray line. The numbers above each box plot represent the number of fermentations in which this genus was detected.

Regarding LAB diversity, Leuconostoc spp. were found at almost all time points of all fermentations, with relative abundances ranging from 0.26% to 18.03%, 2.43% to 24.56%, and 4.60% to 21.22% for days 1, 3, and 30, respectively (Fig. 3). In addition, Lactobacillus spp. were detected in a maximum of 38 fermentations, with a high increase in relative abundance at day 30 compared to the two previous time points, reaching a maximum of 71.96%. Other LAB species were identified as belonging to the genera Lactococcus and Weissella, found in a maximum of 26 and 25 different fermentations, with relative abundances from 0.09% to 49.63% and 0.31% to 39.95%, respectively; this suggests that the spontaneous carrot juice fermentations were driven by a limited number of LAB genera. Furthermore, DNA of Enterobacteriaceae was detected in nearly every sample. The most dominant Enterobacteriaceae ASVs were classified as Citrobacter spp. (detected in 39 fermentations), unclassified Enterobacteriaceae (31 fermentations), Ewingella spp. (39 fermentations), Klebsiella spp. (40 fermentations), Pantoea spp. (36 fermentations), Pectobacterium spp. (11 fermentations), and Yersinia spp. (40 fermentations). In addition, ASVs classified as Pseudomonas spp. were found in a maximum of 7 different fermentations. In contrast to the ASVs belonging to the genus Lactobacillus, these genera all showed a decreasing relative abundance toward day 30. However, detection of the genera of Enterobacteriaceae at later time points in the fermentations was unexpected, since culture-based techniques showed no presence of Enterobacteriaceae on selective VRBG agar medium after, 2, 10, and 30 days of fermentation for LF2, LF1, and the household fermentations, respectively (Fig. 1B to D).

### RNA-based 16S rRNA gene sequencing shifted relative abundances and reduced chloroplast contamination.

**FIG 4:**
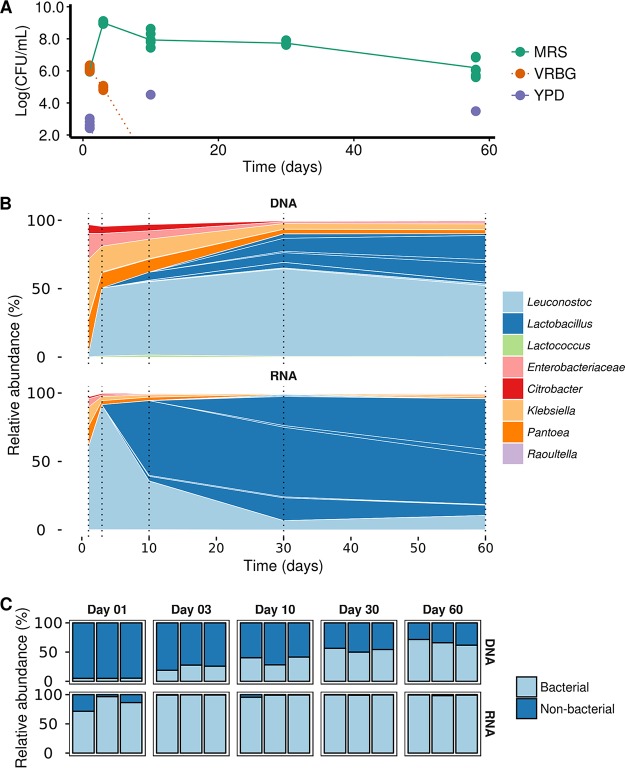
Microbial community dynamics of laboratory carrot juice fermentation LF3. (A) Microbial counts on MRS agar for presumptive LAB, VRBG agar for presumptive Enterobacteriaceae, and YPD agar for presumptive yeasts. (B) Taxonomic profiling based on both DNA and RNA, followed by amplicon sequencing of the V4 region of the 16S rRNA gene. Only the top 20 amplicon sequence variants are shown, and they are colored by their highest unclassified rank. The sample time points are shown with a dashed line. (C) Read distribution of bacterial and nonbacterial sequences after quality control of DNA-based and RNA-based 16S rRNA gene sequencing of the V4 region.

Due to the discrepancy between the culture-dependent and -independent data concerning presumptive Enterobacteriaceae and the low pH values that should inhibit growth of these bacteria, it was hypothesized that the DNA-based 16S rRNA gene sequencing was biased for dead bacterial cells. Thus, to obtain more insights into the presumptive metabolically active bacterial communities, a third laboratory fermentation of carrot juice (LF3) was set up and followed for 2 months (Fig. 4), including an RNA-based 16S rRNA gene sequencing approach.

The progression of LF3 was similar to those of the LF1, LF2, and HF fermentations with respect to viable plate counts (Fig. 4A). High counts of presumptive LAB were found throughout the fermentation, even after 60 days. Presumptive Enterobacteriaceae were present at days 1 and 3 but showed no growth on VRBG agar medium from day 10 on. In addition, some growth on YPD agar was detected at day 1, but, in line with all fermentations described above, this did not persist. Culture-independent data for LF3, obtained by targeting both RNA and DNA, showed the presence of the same taxa, but with large differences in their relative abundances (Fig. 4B). Leuconostoc spp. and Lactobacillus spp. were the most abundant LAB for both approaches. The DNA-based approach showed Leuconostoc spp. to be the dominant LAB throughout this fermentation, with a maximum relative abundance of 64.65% (day 30). In contrast, the RNA-based approach showed Lactobacillus spp. to be the dominant LAB from day 10, with a maximum relative abundance of 92.00% (day 30). Moreover, a higher fluctuation was found with the RNA approach, since the relative abundance of the most abundant Leuconostoc ASV showed a high peak at day 3, which gradually decreased toward day 10. Regarding Enterobacteriaceae, the same taxa were identified using both the RNA- and DNA-based approaches, belonging to five different ASVs (unclassified Enterobacteriaceae, Citrobacter spp., Klebsiella spp., Pantoea spp., and Raoultella spp.), but their relative abundances were much lower in the RNA-based approach (especially from day 10), indicating that they were mostly not metabolically active throughout the fermentation process. Furthermore, a remarkably large difference in nonbacterial read counts (e.g., chloroplasts and mitochondria) was found between the two sampling strategies (Fig. 4C). In fact, the RNA-based approach did greatly reduce chloroplast contamination in this plant-based food fermentation, especially at the earlier time points of fermentation, for which the microbial biomass was low relative to contaminating host sequences, and thus allowed for a higher resolution at similar sequencing depth.

### Phylogenetic placement-based LAB diversity and ecological impact of starter cultures.

Since LAB genera were shown to be the most prominent players, the natural LAB diversity in the spontaneous fermented carrot juices was further explored at the single nucleotide variance level (ASV level) by combining it with a phylogenetic placement strategy. As mentioned above, the most abundant LAB members of LF1, LF2, and all HF fermentations were Leuconostoc spp. and Lactobacillus spp. (Fig. 3). In total, eight different Leuconostoc ASVs (Fig. S2) and 20 different Lactobacillus ASVs (Fig. S3) were identified. The ASV Leuconostoc 2 was the most ubiquitous (Fig. S2), as it was found in 39 out of the 40 fermentations (Table S1). Leuconostoc 1 was identified as the most dominant ASV, with maximum relative abundances ranging from 1.24% to 51.53% (HF36 and HF23, respectively). In addition, the relative abundance of this ASV always increased toward the end of the fermentations. As the pH quickly decreased in these fermentations, this particular ASV probably represented a strain or species with a better tolerance toward a low pH. Indeed, further classification using the EzBioCloud 16S database (25) (Table S1) of both ASVs showed that Leuconostoc pseudomesenteroides was the most probable match for Leuconostoc 1, which is a species known to have a higher tolerance toward acidity than Leuconostoc mesenteroides (26), with L. mesenteroides corresponding to the most probable classification of Leuconostoc 2. Furthermore, Lactobacillus ASVs (Fig. S3) were less ubiquitously found in the fermented carrot juices than Leuconostoc spp., with Lactobacillus 1, Lactobacillus 2, and Lactobacillus 4 as most prevalent ASVs, detected in 23, 23, and 20 out of the 40 fermentations, respectively (Table S1). Moreover, 12 out of 20 Lactobacillus ASVs were uniquely found in a single carrot juice fermentation.

**FIG 5:**
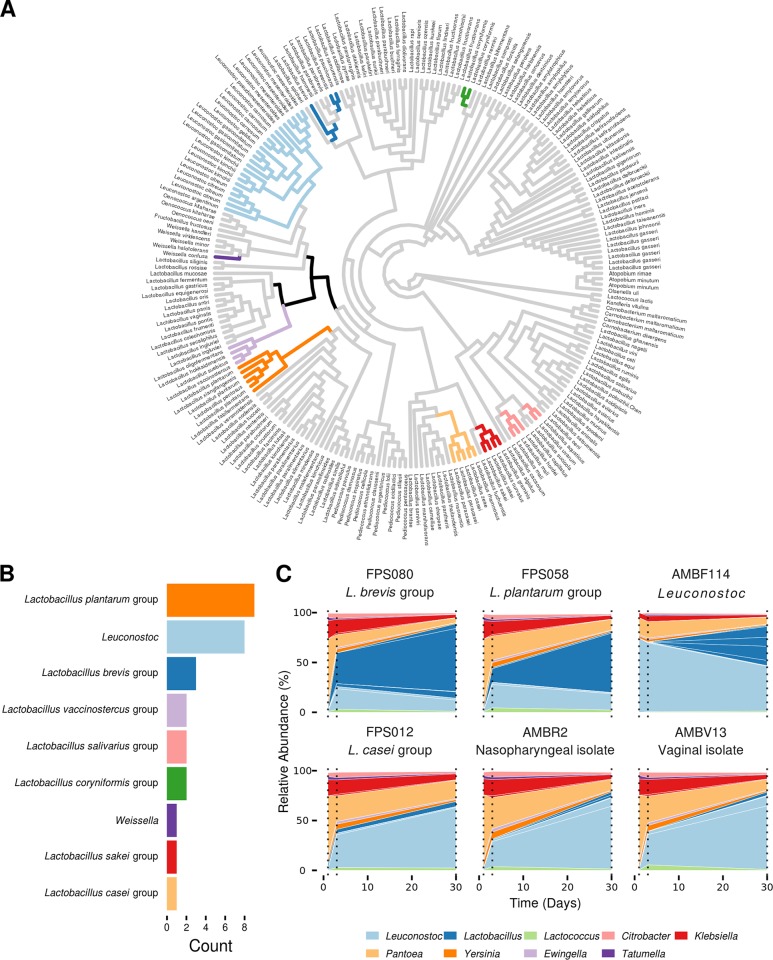
Phylogenetic placement of amplicon sequence variants (ASVs) found in 40 different spontaneous carrot juice fermentations on a 16S rRNA gene phylogenetic tree of the Lactobacillus genus complex and bacterial community dynamics of six starter-culture fermentations. (A) Phylogenetic tree visualized as a cladogram. Branches on which an ASV could be mapped are colored according to the phylogenetic group to which they belong (shown in panel B). Black represents ASVs not classified to a phylogenetic group. Duplications in species names are the result of multiple 16S rRNA gene copies in that particular strain. (B) Number of ASVs per phylogenetic group. Results were filtered to keep only the maximum likelihood weight node for each ASV. (C) 16S rRNA gene sequencing (V4 region) of starter-culture fermentations using six different strains. Only the top 20 ASVs are shown; they are colored by their highest possible classified taxonomic rank. Sampling points are indicated with a dashed line.

Since the genus Lactobacillus contains interesting examples of bacterial niche adaptation with well-supported phylogenetic groups that have lifestyles defined as free-living, nomadic, strictly symbiotic, or vertebrate hosts (27), a phylogenetic placement strategy was performed to further identify the ASVs. Phylogenetic placement of ASVs classified as Lactobacillus, Leuconostoc, and Weissella on a 16S rRNA gene tree of the Lactobacillus genus complex defined by Duar et al. (27) and Zheng et al. (28) showed that these ASVs belonged to 9 out of 28 phylogenetic groups (Fig. 5A and S4). The phylogenetic groups detected in the fermented carrot juices comprised the Lactobacillus brevis group, Lactobacillus casei group, Lactobacillus coryniformis group, Lactobacillus plantarum group, Lactobacillus sakei group, a subpart of the Lactobacillus salivarius group, the Lactobacillus vaccinostercus group, and the genera Leuconostoc and Weissella. The most prevalent phylogenetic groups were identified as L. plantarum group, Leuconostoc, and L. brevis group, with 9, 8, and 3 ASVs placed in these phylogenetic groups, respectively (Fig. 5B).

Strains belonging to these three most abundant phylogenetic groups were subsequently used to perform starter-culture fermentations to assess their influence on LAB diversity of the unpasteurized fermentation process (Fig. 5C). All three strains, AMBF114 (Leuconostoc), FPS058 (L. plantarum group), and FPS080 (L. brevis group), were isolated in one of the above-mentioned fermentations (autochthonous strains). In addition, one other autochthonous strain (FPS012 of the L. casei group) belonging to a less prevalent phylogenetic group, and two allochthonous strains, isolated from human niches (AMBR2 [29] and AMBV13 [ENdEMIC laboratory collection]) were used. While all six starter-culture fermentations showed a similar composition regarding Enterobacteriaceae, a large difference in Lactobacillus presence and abundance was found (Fig. 5C). The fermentations with the three autochthonous strains belonging to the L. brevis group (FPS080), L. plantarum group (FPS058), and Leuconostoc spp. (AMBF114) (Fig. 5C, first row) all showed a higher relative abundance of Lactobacillus spp. than the remaining three fermentations, where almost no lactobacilli were found after 30 days of fermentation (Fig. 5C, second row). In addition, the fermentation inoculated with the Leuconostoc strain showed the highest alpha diversity (Fig. S5) of all starter-culture fermentations at day 30, mainly due to the presence of five different Lactobacillus ASVs.

## DISCUSSION

Spontaneously fermented carrot juice is an example of a food product developed by professional chefs to satisfy the consumers' increasing need for innovative culinary experiences (11). In addition, it forms an interesting model system to explore the impact of anthropogenic pressures as drivers of microbial ecosystems (30). One of the key questions in such a spontaneous fermentation process is how robust the ecosystem will act when applying rather simple external pressures (e.g., addition of salt and storage under semianaerobic conditions in fermentation jars), given the various ingredient sources, the various locations where the fermentations are performed, the different operators, and other environmental factors that may occur.

**FIG 6:**
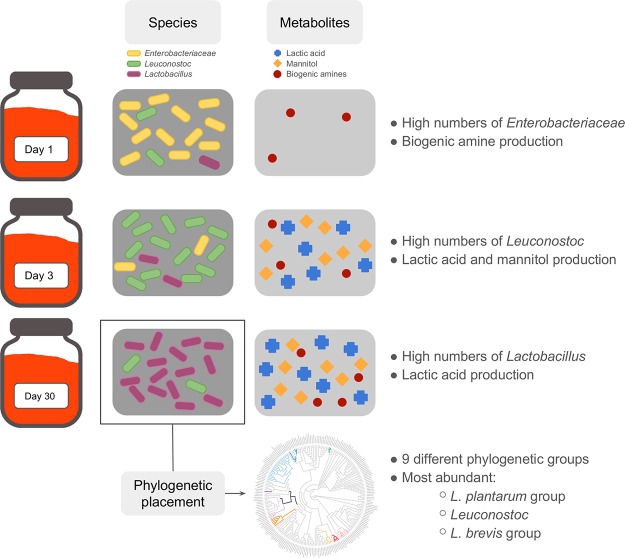
Graphical summary showing the bacterial community dynamics and metabolite production in spontaneous fermented carrot juices, as well as the identification of the main LAB species using phylogenetic placement.

In this study, an active citizen science approach was implemented to explore the robustness and microbial community dynamics of 38 different homemade carrot juice fermentations. Participants were asked to ferment their own carrots (either from their garden, supermarket, or organic origin), in their own jars and kitchens, according to a standard procedure, after attending a workshop. The resulting culture-dependent data of these household fermentations, combined with those of carefully monitored laboratory fermentations, showed that spontaneous carrot juice fermentation is a consistent microbiological process. It is characterized first by the presence of Enterobacteriaceae, followed by a clear shift in microbial abundances, ultimately resulting in a dominance of LAB species (Fig. 6). This drastic shift in microbial communities took place between days 3 and 13 of fermentation, and acidification by the LAB led to a decrease in pH below 4.6, the widely accepted food fermentation safety threshold (22). A fast pH decrease is important to avoid extensive growth of Enterobacteriaceae, which are omnipresent on the surfaces of vegetables and fruits and are thus often found in the early stages of vegetable fermentations (14, 16, 31–33). Many of the Enterobacteriaceae genera identified in the fermentations studied are well documented to be plant or vegetable associated (34–36), where they are described as keystone species, meaning that they play a fundamental role in shaping the overall communities (37). These results thus suggest that the Enterobacteriaceae composition in the early days of spontaneous carrot juice fermentations is shaped by the endogenous carrot microbiome profile, which in turn will be determined by soil or other environmental microorganisms.

High-throughput 16S rRNA gene sequencing revealed the presence of four different LAB genera (Leuconostoc, Lactobacillus, Lactococcus, and Weissella) and a temporal succession of Leuconostoc spp. (Leuconostoc being the most dominant LAB genus at the beginning of the fermentation process) and Lactobacillus spp. (toward the end of the fermentation process) (Fig. 6). Similar dynamics have been shown for the spontaneous fermentation processes of many vegetables, in particular kimchi (38) and sauerkraut (39), during which the LAB communities shift from less-acid-tolerant heterolactic LAB species (e.g., those of Leuconostoc) to more-acid-tolerant homolactic LAB species (e.g., those of Lactobacillus). Such LAB shifts have been shown during other spontaneous fermentation processes as well, for instance, during the fermentation step of wet coffee processing (40).

In contrast to the lack of growth of presumptive Enterobacteriaceae shown with the culture-dependent data, DNA-based 16S rRNA gene sequencing revealed a high relative abundance of Enterobacteriaceae DNA in all spontaneous carrot juice fermentations examined, even at later time points. It was hypothesized that this detection of Enterobacteriaceae was mainly due to the presence of persistent DNA derived from dead bacteria that were alive in the early days of the fermentation when they reached high counts on VRBG agar medium. Similarly, as previously shown for DNA persistence in soil, sediment, and water (41), DNA from dead bacteria appeared to persist during carrot juice fermentations over long time periods, even up to 2 months. Indeed, using an RNA-based 16S rRNA gene sequencing approach, assessing the presumptive active microbial communities of ecosystems (42, 43), confirmed the initial peak of Enterobacteriaceae abundance but showed a drastic reduction upon fermentation, indicating that these Enterobacteriaceae died or at least became metabolically dormant at later time points of the carrot juice fermentations. In addition, higher fluctuations in relative abundances of Leuconostoc spp. and Lactobacillus spp. during carrot juice fermentation showed that the RNA-based method better captured the dynamics of this closed microbial ecosystem. Moreover, this method allowed a significant reduction in contaminating nonbacterial (e.g., chloroplast) sequences, making RNA-based 16S rRNA gene sequencing an ideal strategy for studying microbial ecosystems where host contamination from chloroplasts and mitochondria is expected to be high.

Although the majority of the Enterobacteriaceae did probably not survive the whole duration of the carrot juice fermentations, the high numbers of Enterobacteriaceae at days 1 to 3 posed some health risks, for example, due to the possible production of biogenic amines. Biogenic amines can cause headaches and nausea, or in extreme cases, even anaphylactic shock (11, 44–46). It has been shown that both spoilage microorganisms and LAB are able to produce these compounds (44, 47–51). However, both the European Food Safety Authority (EFSA) and the U.S. Food and Drug Administration (FDA) currently did not define a maximum threshold for general biogenic amine presence in foods (45, 52). The only exception is histamine, which is regulated at a maximum concentration of 200 to 400 mg/kg (EFSA) or 500 mg/kg (FDA) in fish and fish products (45, 49). Regardless, elevated biogenic amine concentrations in fermented foods should be avoided for organoleptic reasons as well. Therefore, a threshold of around 50 to 100 mg/liter has been put forward for cadaverine and putrescine (44). During the present study, cadaverine concentrations around this threshold were measured in the fermented carrot juices, probably produced by the Enterobacteriaceae (Fig. 6). In addition, putrescine and tyramine were found in LF2 simultaneously with an increase in relative abundance of Lactobacillus ASVs. One of these, Lactobacillus 6, is classified as L. brevis, which is known to be able to produce both putrescine and tyramine (53, 54). The fact that biogenic amine production took place during carrot juice fermentation suggested that the development of starter cultures that do not produce biogenic amines (and efficiently outcompete Enterobacteriaceae) could have large potential for a more industrialized carrot (juice) fermentation process.

In the search for suitable starter cultures, the phylogenetic placement strategy described in this study showed that LAB ASVs from different spontaneous carrot juice fermentations were spread all over the Lactobacillus genus complex described by Sun et al. (55) (Fig. 6). All of them were found in clades annotated as either free-living or nomadic lactobacilli, in line with the expected lifestyles proposed before (27, 28). Furthermore, ASVs from both heterofermentative and homofermentative LAB species were found. Fermentations inoculated with strains from the three most prevalent LAB phylogenetic groups showed a high relative abundance in ASVs classified as Lactobacillus after 30 days of fermentation. In contrast, when the strains used originated from a less prevalent phylogenetic group or were allochthonous, the relative abundance of Lactobacillus spp. was low, and Leuconostoc ASVs were the most dominant LAB. Furthermore, fermentation with an autochthonous prevalent Leuconostoc strain increased the general alpha diversity as well as the diversity in Lactobacillus ASVs, resulting in a more diverse LAB-dominated man-made ecosystem. As general prevalence was thus found to be an important parameter for starter-culture development, the most ubiquitous ASVs were identified here as Lactobacillus 1, Lactobacillus 2, and Lactobacillus 4. Since phylogenetic placement showed that all three of them belonged to the L. plantarum group, future work regarding starter-culture development could thus focus on isolates belonging to this phylogenetic group. In general, L. plantarum starter-culture strains are often used to control vegetable fermentation processes (3, 56).

To summarize, the results presented in this study showed that spontaneous fermented carrot juice, a novel food product, is dominated by a considerable number of LAB belonging to different species of the Lactobacillus genus complex, making it a useful LAB-rich microbial model ecosystem. The spontaneous carrot juice fermentation process appeared to be fairly robust and repeatable, as the main genera were identified in most of the fermentations performed in the framework of an active citizen science project. However, this food product could benefit from the development of starter cultures to avoid high numbers of Enterobacteriaceae and/or high biogenic amine concentrations. Finally, this study highlighted the potential of RNA-based 16S rRNA gene sequencing to study the presumptive active microbial communities, with the added benefit of avoiding host (e.g., chloroplast or mitochondrial) read contamination.

## MATERIALS AND METHODS

### Household fermentations, microbial plating, pH measurement, and total DNA extraction.

A citizen science project was set up to carry out spontaneous fermentations of juices from carrots (Daucus carota), so-called household fermentations (HF). Therefore, 41 participants from the region of Antwerp (Belgium) were asked to perform three carrot juice fermentations at home, applying preset guidelines. Briefly, these guidelines comprised (i) the production of 3 liters of carrot juice, to be divided over 3 airtight jars of 1 liter each; (ii) the addition of 2.5% (mass/vol) salt, following a protocol drafted together with Michelin star chef Kobe Desramaults (Dranouter, Belgium); and (iii) storage in a temperature-stable room away from direct sunlight. At three time points, i.e., after 1, 3, and 30 days of fermentation, one jar was withdrawn for sampling. Samples were transported to the laboratory chilled and underwent selective plating on the following three agar media: de Man-Rogosa-Sharpe agar (MRS; Carl Roth, Karlsruhe, Germany) supplemented with cycloheximide (final concentration, 0.1 g/liter), violet-red-bile-glucose agar (VRBG; Carl Roth) supplemented with cycloheximide (final concentration, 0.1 g/liter), and yeast extract-peptone-dextrose (YPD) agar (Carl Roth) supplemented with chloramphenicol (final concentration, 0.1 g/liter). Incubations took place at 37°C for 1 day (VRBG and YPD) or 2 days (MRS), after which the number of CFU was determined. The pH was measured with a FiveEasy FE20 pH meter (Mettler Toledo, Columbus, OH), and total DNA was extracted using the Mo Bio PowerSoil DNA isolation kit (Mo Bio Laboratories, Carlsbad, CA).

### Laboratory fermentations, microbial plating, pH measurement, and total DNA extraction.

Carrots, stored for approximately half a year in soil at 4°C (LF1) or freshly harvested (LF2), were washed and pressed by means of a centrifugal juicer to obtain 33 liters of carrot juice. After the addition of salt (2.5% [mass/vol]) and mixing, the carrot juice was distributed in 33 airtight jars of 1 liter each. In total, 11 time points were sampled over a 2-month period, and at each time point, three jars were withdrawn. Immediately after sampling, the pH of the fermenting juice was measured (FiveEasy FE20; Mettler Toledo). Samples were subjected to selective plating on three different agar media, as described above. In addition, for each sample, total DNA was extracted from 750 μl of carrot juice using the Mo Bio PowerSoil DNA isolation kit (Mo Bio Laboratories) prior to 16S rRNA gene sequencing. Additional samples were frozen at −20°C for metabolite target analysis.

### High-throughput DNA-based 16S rRNA gene sequencing.

Sequencing of 16S rRNA gene amplicons was performed using a dual-index strategy (57). Briefly, DNA samples were subjected to a barcoded PCR assay, in duplicate, amplifying the V4 region of the 16S rRNA gene using a Phusion high-fidelity DNA polymerase (New England BioLabs, Ipswich, MA). PCR amplicons were purified, the concentrations normalized using the SequalPrep normalization kit (Invitrogen, Life Technologies, Grand Island, NY), and pooled to obtain an amplicon library. As a final quality control, this pooled amplicon library was subjected to electrophoresis on a 0.8% (mass/vol) agarose gel, after which the amplicons were cut out and purified with the NucleoSpin gel and PCR cleanup kit (Macherey-Nagel, Düren, Germany). Finally, the amplicon library concentration was measured using the Kapa library quantification kit (Kapa Biosystems, Wilmington, MA), diluted to 2 nM, spiked with 10% (vol/vol) PhiX DNA (Illumina, San Diego, CA), and sequenced on the Illumina MiSeq platform using 2 × 250 cycles at the Center of Medical Genetics Antwerp (University of Antwerp, Antwerp, Belgium).

### Sequence analysis.

The raw sequencing data were processed using the DADA2 package (23) in the R environment version 3.4.0 (58), following the DADA2 standard operation protocol. In short, sequences with at least one ambiguous base, reads containing the lowest possible quality score of 2, and reads with more than two “expected errors” were discarded. Following trimming of the first 10 bp, the forward reads were truncated at position 240, whereas the reverse reads were truncated at position 160. Next, sample inference (DADA2's core algorithm) was performed, followed by read merging. A sequence table containing all amplicon sequence variants (ASVs) was constructed, chimeras were removed, and taxonomy was assigned using RDP version 14 (59). The sequence table and taxonomy were then imported into the Phyloseq package version 1.19.1 (60). Subsequent analysis included the removal of contaminating DNA, calculation of alpha- and beta-diversity metrics, and plotting of taxonomic profiles after clustering based on average linkage hierarchical clustering of the Bray-Curtis dissimilarity matrix of all day 30 samples using Vegan version 2.4-4 (61). Further classification of ASVs of interest was performed using the EzBioCloud 16S database (25). A full version of the pipelines described can be found as an R markdown and HTML file (https://github.com/swuyts/Spontaneous-carrot-juice-fermentation).

### Phylogenetic placement.

All Lactobacillus genus complex genomes (55) were retrieved from the NCBI genome database (27 September 2017). Subsequently, the 16S rRNA genes of all assemblies were extracted using barrnap and aligned with MAFFT (70), and using this alignment, a phylogenetic tree was built with RAxML (version 8.2.11) (62). Multiple 16S rRNA gene copies were kept for completeness. Next, all sequences of ASVs classified as belonging to the Lactobacillus genus complex were added to the alignment using MAFFT and were placed on the phylogenetic tree using the evolutionary placement algorithm implemented in RaxML. As additional parameters, a minimum likelihood weight of at least 50% of that of the maximum placement weight for the ASVs and a maximum of 100 placements was used. Finally, visualization was done with ggtree (63).

### Starter-culture fermentations.

Six different strains were used as starter cultures for the fermentation of carrot juice. Four of these strains (Leuconostoc mesenteroides AMBF114, Lactobacillus paracasei FPS012, Lactobacillus plantarum group FPS058, and Lactobacillus brevis FPS080) were isolated from one of the above-described fermentations. The two remaining strains, AMBR2 (Lactobacillus casei) and AMBV13 (Lactobacillus gasseri), were isolated from the human nasopharyngeal tract (29) and the human vagina (ENdEMIC laboratory collection), respectively. All strains were isolated from MRS agar, and their DNA was extracted using the NucleoSpin 96 tissue kit (Macherey-Nagel), with an extra lysis step using lysozyme (20 mg/ml) and mutanolysin (100 U/ml). The full 16S rRNA gene was amplified using the universal bacterial primers 27F (65) and 1492R (66) and subsequently sequenced with the same primer set, using Sanger sequencing, at the Genetic Service Facility (Vlaams Instituut voor Biotechnologie [VIB], Antwerp, Belgium). Sequences were processed using Geneious 8 (67) and identified using the EzBioCloud 16S database (25). For the starter-culture fermentations, these strains were first precultured in MRS medium, after which an overnight culture was diluted to 10^8^ CFU/ml, washed with phosphate-buffered saline, and finally resuspended in fresh carrot juice. In addition, 18 liters of carrot juice was prepared, as described above, and divided over 18 different 1-liter jars. Each starter culture was added to three jars, allowing for the analysis of three time points (days 1, 3, and 30) per strain. Selective plating, DNA extraction, high-throughput DNA-based 16S rRNA gene sequencing, and sequence analysis were performed as described above.

### Metabolite target analysis.

Substrates and metabolites were analyzed as described before (33). High-performance liquid chromatography with refractive index detection (HPLC-RI) was used to determine the concentrations of lactic acid and acetic acid. In addition, high-performance anion-exchange chromatography with pulsed amperometric detection (HPAEC-PAD) was used to measure glucose, fructose, sucrose, and mannitol concentrations. Preparation of the samples and further processing of the data were carried out as described previously (68). Biogenic amine concentrations were measured by means of ultraperformance liquid chromatography coupled to tandem mass spectrometry (UPLC-MS/MS), as described previously (69).

### High-throughput RNA-based 16S rRNA gene sequencing.

A third spontaneous laboratory fermentation (LF3) was carried out to assess the active microbial community. Freshly harvested carrot juices were washed and pressed by means of a centrifugal juicer to obtain 15 liters of carrot juice. Similar to the two other laboratory fermentations, salt was added (2.5% [mass/vol]), and the carrot juice was divided among 15 airtight jars of 1 liter each. In total, five time points were sampled over a 2-month period, and at each time point, three jars were withdrawn. The pH was measured, and selective plating and DNA-based 16S rRNA gene sequencing were performed, as described above. In addition, environmental RNA was extracted using the RNeasy microbiome kit (Qiagen, Hilden, Germany), according to the manufacturer's instructions. An additional DNase treatment was performed using Turbo DNA-*free* (Thermo Fisher Scientific, Waltham, MA) to remove leftover DNA prior to cDNA synthesis with the Superscript III reverse transcriptase (Thermo Fisher Scientific) and the 806R 16S rRNA primer. Finally, the cDNA was sequenced using the above-mentioned sequencing pipeline, and the sequences were analyzed as described above.

### Accession number(s).

Sequencing data are available at the European Nucleotide Archive with the accession number PRJEB15657.

## Supplementary Material

Supplemental material
