# Supplementary material for: Carrot Juice Fermentations as Man-Made Microbial Ecosystems Dominated by Lactic Acid Bacteria
Source: Appl Environ Microbiol. 2018 May 31;84(12):e00134-18. doi: 10.1128/AEM.00134-18 (PMC5981062; doi:10.1128/AEM.00134-18)
Supplement: Supplemental material [file supp_84_12_e00134-18__index.html]

Supplemental material 

# Carrot Juice Fermentations as Man-Made Microbial Ecosystems Dominated by Lactic Acid Bacteria

## Supplemental material

- Supplemental file 1 -

  *Leuconostoc* and *Lactobacillus* amplicon sequence variants (ASVs) from carrot juice fermentations, their occurrence, maximum relative abundance, and identification using the EzBioCloud 16S database, and the number of differential versus total nucleotides (Table S1); alpha-diversity metrics of all laboratory and household carrot juice fermentations examined after 16S rRNA gene sequencing of the V4 region and data processing using DADA2 (Fig. S1); 16S rRNA gene sequencing taxonomic profiles showing all *Leuconostoc* (Fig. S2) and *Lactobacillus* (Fig. S3) ASVs of laboratory carrot juice fermentations 1 and 2 and household carrot juice fermentations; phylogenetic placement of ASVs on a 16S rRNA gene phylogenetic tree of the *Lactobacillus* genus complex visualized for all LAB ASVs (Fig. S4); alpha-diversity metrics of all starter-culture fermentations after 16S rRNA gene sequencing of the V4 region and data processing using DADA2 (Fig. S5).

  PDF, 7.6M
