# Supplementary material for: Carrot Juice Fermentations as Man-Made Microbial Ecosystems Dominated by Lactic Acid Bacteria
Source: Appl Environ Microbiol. 2018 May 31;84(12):e00134-18. doi: 10.1128/AEM.00134-18 (PMC5981062; doi:10.1128/AEM.00134-18)
Supplement: Supplemental material [file AEM.00134-18_zam012188562s1.pdf]

SUPPLEMENTAL MATERIAL

Table S1. Leuconostoc and Lactobacillus amplicon sequence variants (ASVs) from the carrot juice fermentations (laboratory, LF1 and LF2; household, HF), their occurrence, maximum relative abundance, identification using EzBioCloud 16S database (1), and the number of differential versus total nucleotides.

ASV	Occurrence (# fermentations)	Maximum relative abundance (%)	EzBiocloud identification	Differential / Total nucleotides
Leuconostoc				
Leuconostoc 1	20	51,53	*Leuconostoc pseudomesenteroides*	0/233
Leuconostoc 2	39	37,00	*Leuconostoc mesenteroides*	0/233
Leuconostoc 3	35	39,62	*Leuconostoc gelidum,*<br>*Leuconostoc myukkimchii,*<br>*Leuconostoc inhae*	0/233
Leuconostoc 4	9	14,86	*Leuconostoc rapi,*<br>*Leuconostoc kimchii*	0/233
Leuconostoc 5	14	10,05	*Leuconostoc gelidum subsp. gasicomitatum*	0/233
Leuconostoc 6	7	6,05	*Leuconostoc lactis,*<br>*Leuconostoc palmae,*<br>*Leuconostoc holzapfelii,*<br>*Leuconostoc citreum*	0/233
Leuconostoc 7	6	4,78	*Leuconostoc gelidum,*<br>*Leuconostoc myukkimchii,*<br>*Leuconostoc inhae*	1/233
Leuconostoc 8	1	1,24	*Leuconostoc carnosum*	0/233
Lactobacillus				
Lactobacillus 1	23	70,55	*Lactobacillus xiangfangensis,*<br>*Lactobacillus modestisalitolerans*	1/233
Lactobacillus 2	23	63,01	*Lactobacillus herbarum*	1/233
Lactobacillus 3	9	50,50	*Lactobacillus mudanjiangensis*	1/233
Lactobacillus 4	20	35,8	*Lactobacillus pentosus,*<br>*Lactobacillus fabifermentans,*<br>*Lactobacillus paraplantarum,*<br>*Lactobacillus plantarum*	0/233
Lactobacillus 5	7	32,91	*Lactobacillus curvatus,*<br>*Lactobacillus sakei*	0/233
Lactobacillus 6	6	9,43	*Lactobacillus brevis*	0/233
Lactobacillus 7	1	23,62	*Lactobacillus pentosus,*<br>*Lactobacillus fabifermentans,*<br>*Lactobacillus paraplantarum,*<br>*Lactobacillus plantarum*	1/233
Lactobacillus 8	1	21,18	*Lactobacillus uvarum,*<br>*Lactobacillus aquaticus*	0/233
Lactobacillus 9	1	13,79	*Lactobacillus coryniformis*	0/233
Lactobacillus 10	2	13,14	*Lactobacillus nejiangensis*	0/233
Lactobacillus 11	1	9,94	*Lactobacillus coryniformis*	0/233
Lactobacillus 12	1	9,66	*Lactobacillus pentosus,*<br>*Lactobacillus fabifermentans,*<br>*Lactobacillus paraplantarum,*<br>*Lactobacillus plantarum*	1/233
Lactobacillus 13	1	8,69	Lactobacillus vaccinostercus,<br>Lactobacillus wasatchensis,<br>*Lactobacillus sp.* SW201	0/233
Lactobacillus 14	1	7,91	*Lactobacillus pentosus,*<br>*Lactobacillus fabifermentans,*<br>*Lactobacillus paraplantarum,*<br>*Lactobacillus plantarum*	1/233
Lactobacillus 15	1	2,61	*Lactobacillus xiangfangensis,*<br>*Lactobacillus modestisalitolerans*	1/233
Lactobacillus 16	3	6,14	*Lactobacillus hordei,*<br>*Lactobacillus mali*	0/233
Lactobacillus 17	1	1,30	*Lactobacillus paucivorans*	1/233
Lactobacillus 18	1	0,89	*Lactobacillus casei, Lactobacillus paracasei*	0/233
Lactobacillus 19	1	1,70	*Lactobacillus herbarum*	2/233
Lactobacillus 20	1	0,33	*Lactobacillus paucivorans*	2/233

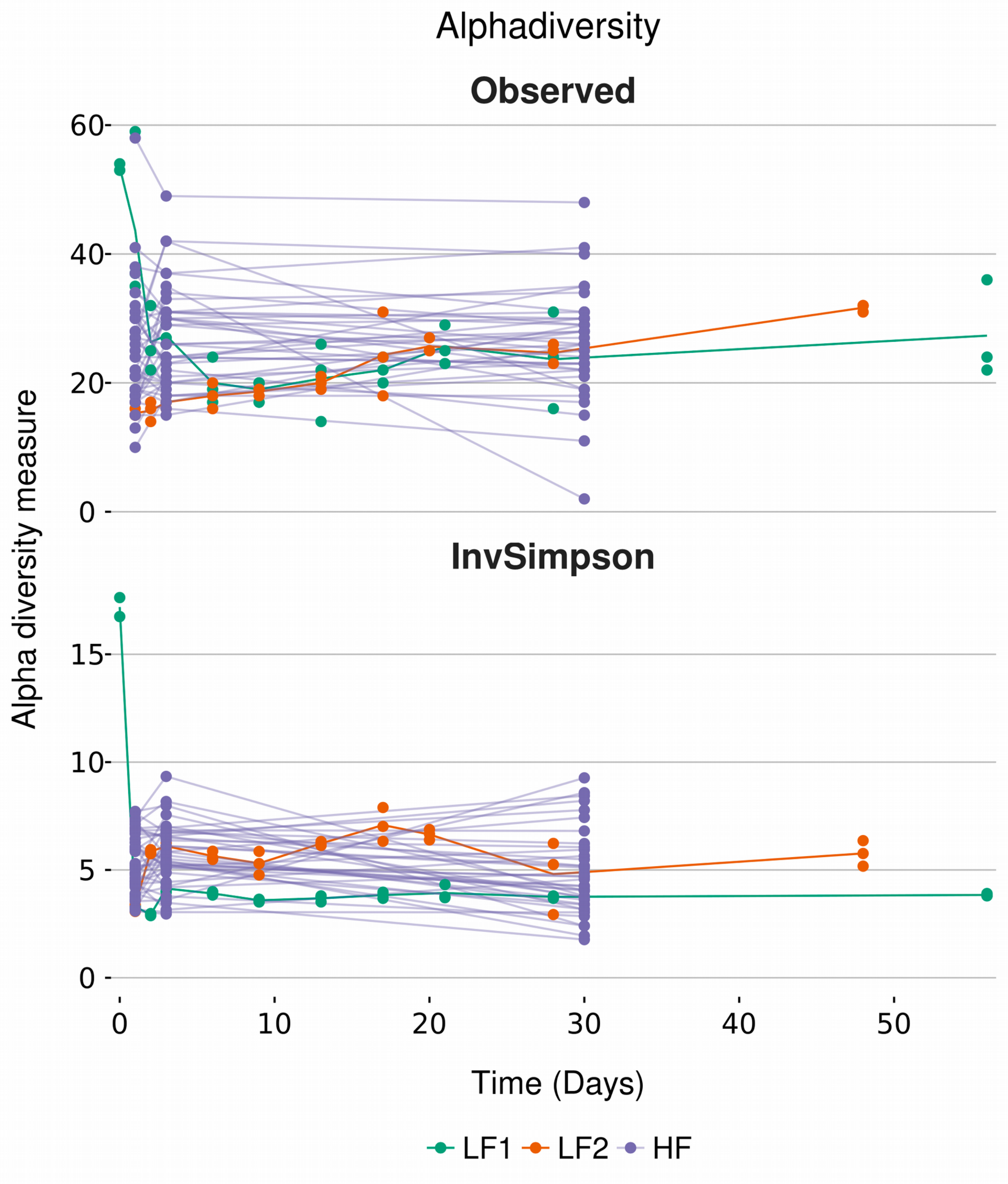

Figure S1. Alpha diversity metrics of all laboratory (LF1 and LF2) and household (HF) carrot juice fermentations examined after 16S rRNA gene sequencing of the V4 region and data processing using DADA2 (2).

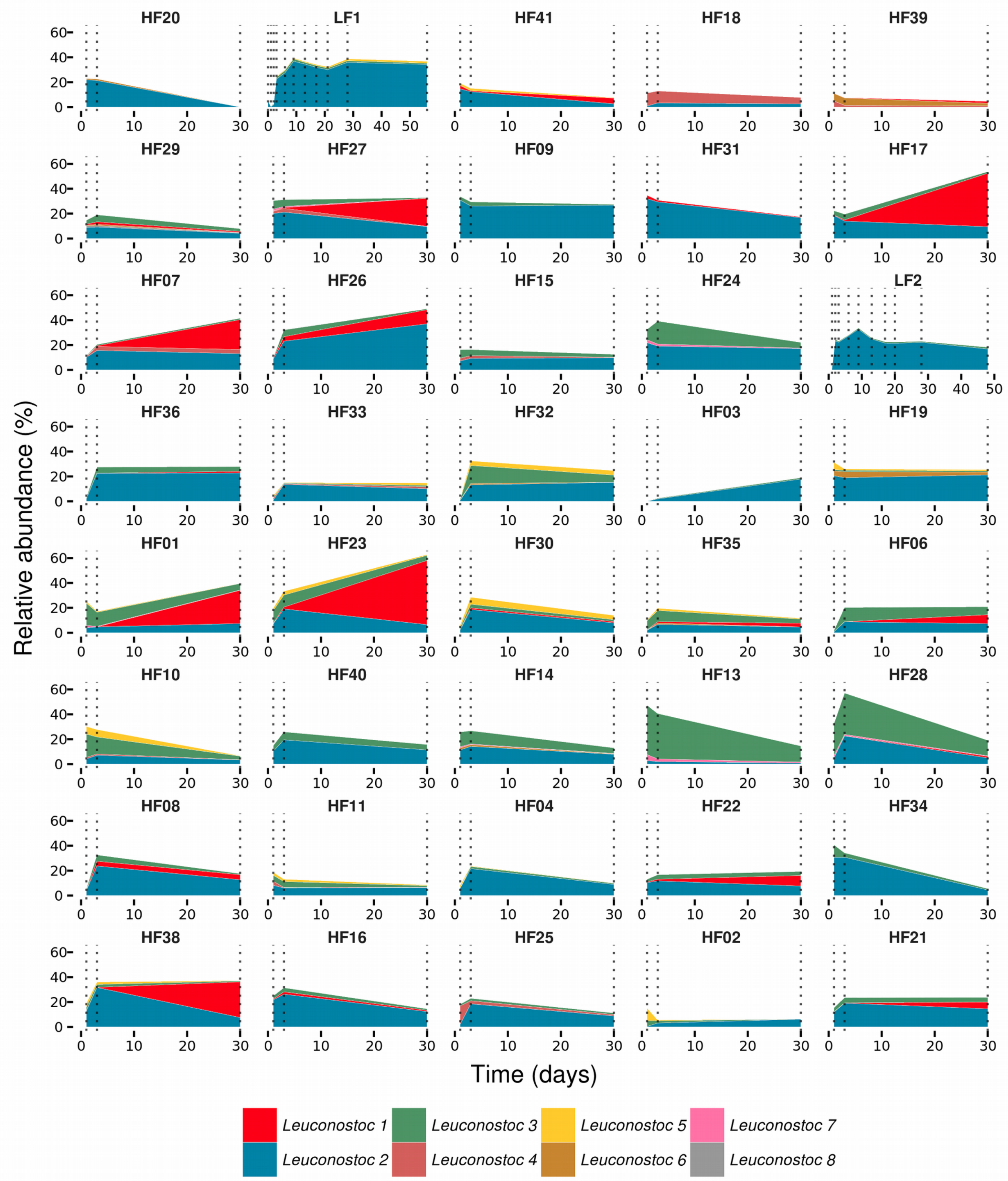

Figure S2. 16S rRNA gene sequencing taxonomic profiles showing all Leuconostoc ASVs of the laboratory carrot juice fermentation 1 (LF1), laboratory carrot juice fermentation 2 (LF2) and the household carrot juice fermentations (HF01–HF41). Sampling points are indicated with a dashed line. The order of appearance of the fermentations is based on hierarchical clustering of the Bray-Curtis dissimilarity matrix of all day-30 samples.

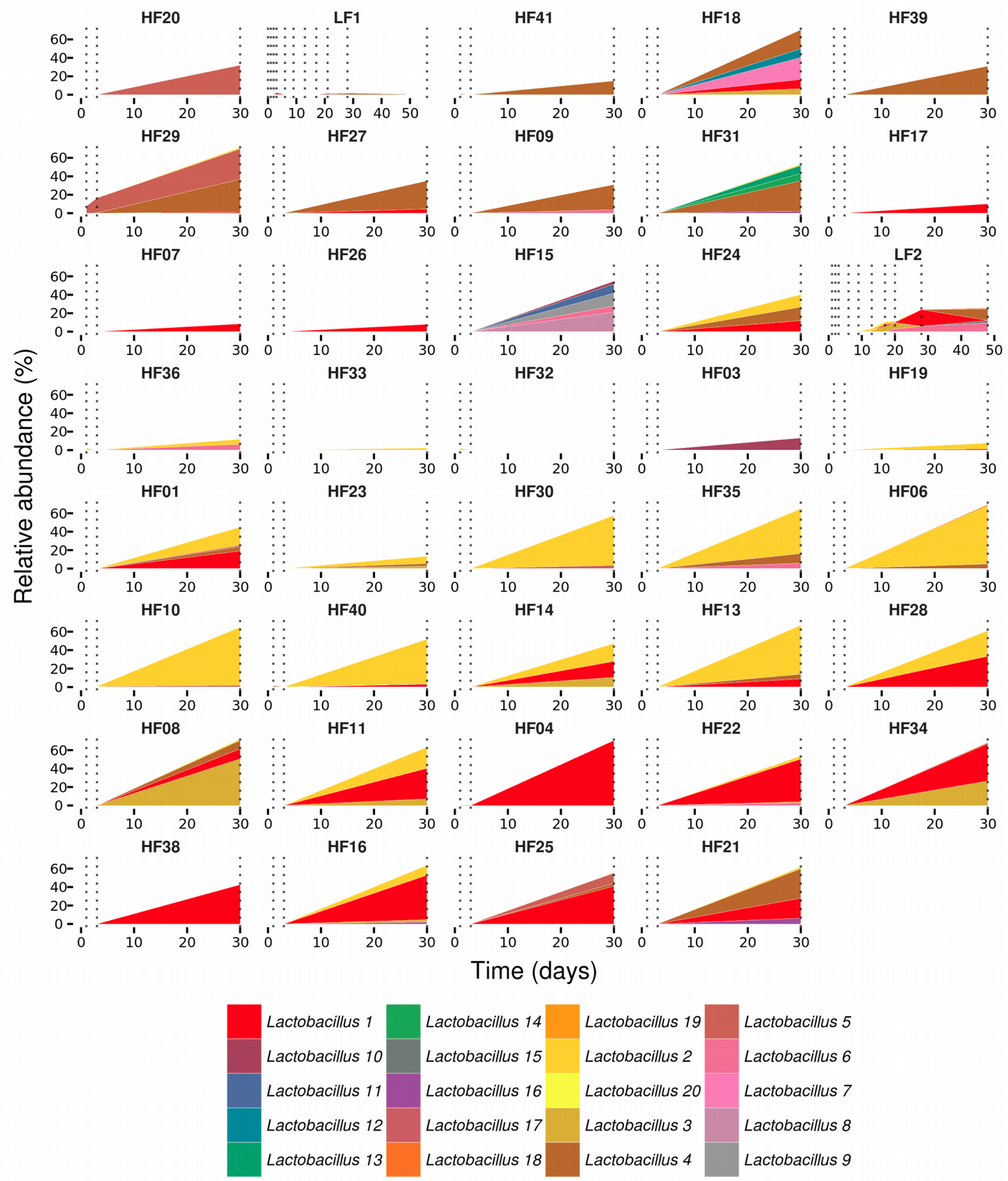

Figure S3. 16S rRNA gene sequencing taxonomic profiles showing all Lactobacillus ASVs of the laboratory carrot juice fermentation 1 (LF1), laboratory carrot juice fermentation 2 (LF2) and the household carrot juice fermentations (HF01–HF41). Sampling points are indicated with a dashed line. The order of appearance of the fermentations is based on hierarchical clustering of the Bray-Curtis dissimilarity matrix of all day-30 samples.

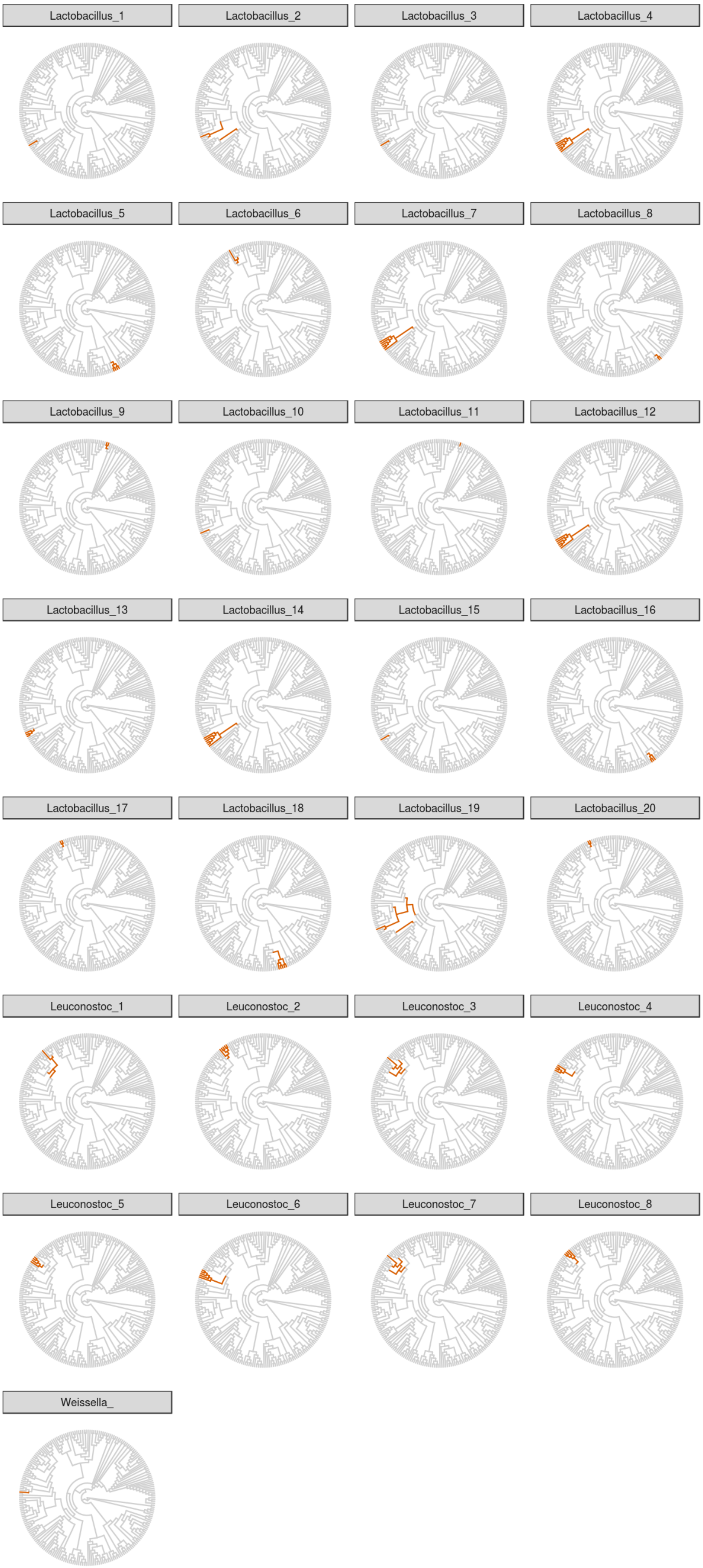

Figure S4. Phylogenetic placement of ASVs on a 16S rRNA gene phylogenetic tree of the Lactobacillus genus complex visualized for all LAB ASVs. The phylogenetic tree is visualized as a cladogram. Branches on which an ASV could be mapped are colored in orange.

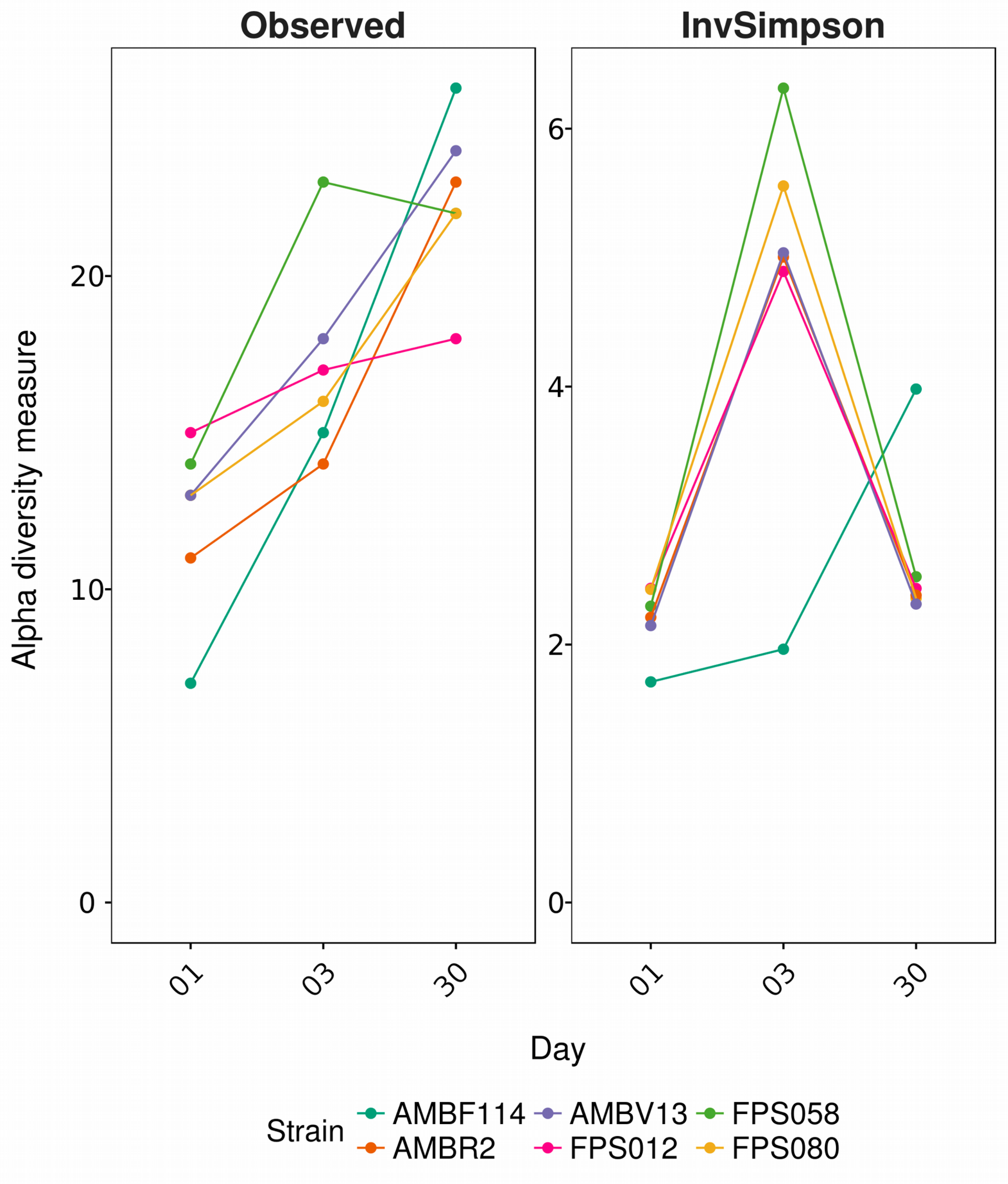

Figure S5. Alpha diversity metrics of all starter-culture fermentations after 16S rRNA gene sequencing of the V4 region and data processing using DADA2 (2).

References

1. Yoon SH, Ha SM, Kwon S, Lim J, Kim Y, Seo H, Chun J. 2017. Introducing EzBioCloud: A taxonomically united database of 16S rRNA gene sequences and whole-genome assemblies. Int J Syst Evol Microbiol 67:1613–1617.

2. Callahan BJ, McMurdie PJ, Rosen MJ, Han AW, Johnson AJA, Holmes SP. 2016. DADA2: High-resolution sample inference from Illumina amplicon data. Nat Methods 13:581–583.
